# Genome Sequence of Staphylococcus epidermidis AUH4567, a Clinical Isolate from an Infected Central Venous Catheter

**DOI:** 10.1128/MRA.01254-20

**Published:** 2021-03-25

**Authors:** Rikke Louise Meyer, Sandra M. Skovdal, Ian P. G. Marshall, Lars Schreiber, Niels Nørskov-Lauritsen, Nis Pedersen Jørgensen

**Affiliations:** a Interdisciplinary Nanoscience Center, Aarhus University, Aarhus, Denmark; b Department of Biology, Aarhus University, Aarhus, Denmark; c Department of Infectious Diseases, Aarhus University Hospital, Aarhus, Denmark; d Department of Clinical Microbiology, Aarhus University Hospital, Aarhus, Denmark; Georgia Institute of Technology

## Abstract

Staphylococcus epidermidis is a common cause of implant-associated infections, and this is related to its ability to form biofilms. Strain-to-strain variability in biofilm formation is likely caused by genetic differences. Here, we present a draft genome of S. epidermidis AUH4567, which was isolated from a central venous catheter infection.

## ANNOUNCEMENT

Staphylococcus epidermidis is the bacterial species most frequently isolated from implant-associated infections, partly due to its abundance on human skin and its ability to form biofilms (1). The mechanisms by which S. epidermidis attaches to and forms biofilm on, for example, implanted devices vary greatly among clinical strains, and knowledge about the genetics underlying biofilm formation is lacking due to abundant methylation-restriction systems that challenge genetic manipulation. Comparative genomics offers another avenue for studying the genetic differences that result in different modes of biofilm formation among S. epidermidis strains.

S. epidermidis AUH4567 was isolated in 2010 at Aarhus University Hospital from blood cultures and a central venous catheter from a patient who had recently undergone major invasive surgery. The isolate was streaked onto 5% blood agar plates (SSI Diagnostic, Denmark) and identified by matrix-assisted laser desorption ionization–time of flight mass spectrometry (MALDI-TOF MS). Subsequently, biofilms grown in tryptic soy broth (TSB) (Sigma-Aldrich, Denmark) under flow conditions showed that the strain formed substantially more biofilm than other clinical S. epidermidis isolates in our laboratory. Thus, the purpose of genome sequencing of this strain was to investigate the presence of biofilm-associated genes and to relate them to the observed phenotype. S. epidermidis AUH4567 was isolated from a human subject who cannot be identified from any material in this manuscript. Our research was carried out in accordance with the WMA Declaration of Helsinki (Ethical Principles for Medical Research Involving Human Subjects [https://www.wma.net/policies-post/wma-declaration-of-helsinki-ethical-principles-for-medical-research-involving-human-subjects/]).

Prior to DNA isolation, S. epidermidis AUH4567 was streaked onto tryptic soy agar (Sigma-Aldrich) and incubated for 24 h at 37°C. Next, a single colony was picked and cultivated in TSB in Erlenmeyer flasks at 37°C for 16 h at 180 rpm. DNA extraction was performed with a blood and cell culture DNA midiprep kit (catalog number 13343; Qiagen) according to the manufacturer’s instructions, using 0.15 mg lysostaphin and 0.15 mg lysozyme in the lysis step. The genome was sequenced using the Illumina MiSeq platform with a paired-end 300-bp MiSeq reagent kit version 3. The sequencing yielded 4,455,704 reads and a total of 1.08 billion bp. The reads were inspected for data quality using FastQC version 0.11.5 (http://www.bioinformatics.babraham.ac.uk/projects/fastqc). Default parameters were used for all software unless otherwise noted. Reads were trimmed using Trimmomatic version 0.36 (2), removing bases outside positions 20 to 289, clipping the read if the quality score of a 4-bp sliding window was below 20, removing Illumina adapters, and omitting reads of <100 bp. The trimmed reads had a total length of approximately 763 million bp, resulting in coverage of 300×. Trimmed reads were then assembled using SPAdes version 3.9.0 (3) with the following parameters: --careful -k 21,33,55,77,99,127 --cov-cutoff auto. The quality of the assembly was then assessed by QUAST version 4.3. A contamination check was performed by CheckM version 1.0.7, using 20 reference genomes for S. epidermidis from the CheckM database (version 2015). The analysis indicated no contamination and 99.76% completeness of the genome. Nevertheless, a decontamination step was performed by removing contigs with <15× coverage (5% of the average coverage), resulting in removal of 23 contigs that were <1,000 bp long. The draft genome was 2,541,034 bp long, consisted of 53 contigs, and had a GC content 34.2%. The *N*_50_ value was 167,030 bp, and the contig coverage was 305×. The genome was annotated using the Prokaryotic Genome Annotation Pipeline (PGAP) (4). The strain has an average nucleotide identity (ANI) of 99.5% with respect to Staphylococcus epidermidis ATCC 14990 (GenBank accession number GCF_006094375.1), as determined by FastANI version 1.32. An ANI value of >95% typically indicates that two strains belong to the same species (5).

Orthologs of biofilm-associated genes were identified by BLAST using a bitscore ratio cutoff value of 0.3. The surface-bound adhesins *gehD* (GenBank accession number AAC67547.1), *sdrF* (AAF72509.1), *embp* (AAM51173.1), and *atlE* (NP_765436.1) and the *icaADBC* operon (AAQ88121.1) for polysaccharide production were present in the genome, while the adhesins *aap* (ADK78216.1), *sdrG* (AAF72510.1), and *sdrH* (EJE36495.1) were absent. All response regulators known to affect biofilms, i.e., *sarA* (EFA88767.1), *agrA* (ACN65708.1), *agrB* (ACN65705.1), *agrC* (ACN65707.1), and *agrD* (ACN65706.1), were present.

### Data availability.

This whole-genome shotgun project was deposited in DDBJ/ENA/GenBank under the accession number MVFV00000000. The version described in this paper is the first version, MVFV01000000. The GenBank assembly accession number is GCA_002156425.1. The genome was also deposited in the JGI Genomes OnLine Database (GOLD) (accession number Gp0224902).
